# Performance of PEO/Polymer Coatings on the Biodegradability, Antibacterial Effect and Biocompatibility of Mg-Based Materials

**DOI:** 10.3390/jfb13040267

**Published:** 2022-11-30

**Authors:** Arash Fattah-alhosseini, Razieh Chaharmahali, Armin Rajabi, Kazem Babaei, Mosab Kaseem

**Affiliations:** 1 Department of Materials Engineering, Bu-Ali Sina University, Hamedan 65178-38695, Iran; 2 Department of Mechanical and Manufacturing Engineering, Faculty of Engineering and Built Environment, Universiti Kebangsaan Malaysia, Bangi 43600, Malaysia; 3 Department of Nanotechnology and Advanced Materials Engineering, Sejong University, Seoul 05006, Republic of Korea

**Keywords:** Mg alloy, PEO, polymer coating, corrosion, biocompatibility

## Abstract

Magnesium (Mg) alloys have recently attracted attention in biomedicine as biodegradable materials with non-toxic degradable products. Such compounds have become a frontier in the study of biodegradable materials because of their remarkable biomechanical compatibility and superior biocompatibility. The use of Mg-based implants reduces the negative consequences of permanent biological implants by eliminating the necessity for biomaterial surgery following the healing process. However, the quick deterioration, formation of considerable gas of hydrogen volumes and a rise in the body environment pH are obstacles in the application of Mg as an implant material. Hence, compelling advances for erosion resistance and biocompatibility of magnesium and its alloys are noteworthy. Surface modification may be a practical approach because it improves the erosion resistance compared with extensive preparation of a treated surface for progressed bone recovery and cell attachment. Coating produced by plasma electrolytic oxidation (PEO) seems a compelling method in order to enhance magnesium and the properties of its alloys. PEO-formed coatings cannot provide long-term protection in the physiological environment due to their porous nature. Thus, a polymer coating is applied on the porous PEO-formed coating, which is steadily applied on the surface. Polymer coatings improve the biocompatibility properties of Mg and its alloys and increase corrosion resistance. In this article, the most recent advancements in PEO/polymer composite coatings are reviewed, and the biocompatibility of such coatings is examined.

## 1. Introduction

Metal implants are mostly utilized for curing fractures in the bone. Alloys of Co and Ti and stainless steel are widely applied in commercial materials for bone implants. Metals for long-term, steady and load-bearing inserts are selected by considering their remarkable ductility and high fracture resistance. Implants should have biocompatibility and mechanical properties that are compatible with the bone. Regarding metal implants, dangerous metal ions made by wear and corrosion can lead to apoptosis of cells, inflammation and other reactions of the tissue [1,2,3,4]. Biodegradable metals (BMs) have been developed to address these concerns and to prevent subsequent surgery for removing metallic implants after tissue restoration [5,6]. Magnesium (Mg), zinc (Zn) and iron (Fe) are the most basic and earliest BMs. As the first and foremost requirement of any implant material, biocompatibility must be defined as a material’s ability to perform with an appropriate host response in a specific application [7]. Amongst these metals, Mg and its alloys possess high strength, low elastic modulus and low density [8,9,10]. Their elastic moduli are the same as that of the human bone, thereby decreasing the impact of stress-shielding. Furthermore, the use of biodegradable implants reduces the need for another operation for implant removal, which saves money for the health system while also benefiting patients [11]. Degradable implants cause tissue damage due to a slow load transfer from the implant to the tissue. Mg, as an integral part of bone structure and the fourth most abundant cation in the human body, has high biocompatibility, making it one of the best choices for temporary implants [12].

**Figure 1:**
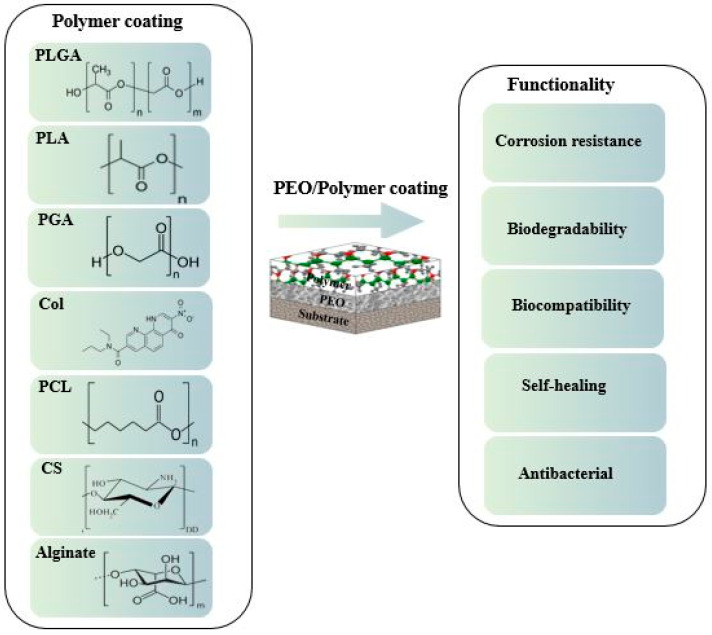
Chemical structure of representative polymeric coatings on magnesium alloys.

Thus, Mg-based BMs have been utilized in a vast number of biological uses, such as inner medical tools for fixation (e.g., bone, pins and screws) and scaffolds of tissue engineering [13,14]. Mg and its alloys differ from other biomaterials because of their mechanical and physical characteristics that are compatible with the human bone [15]. Nevertheless, the use of Mg as material for embedding within pharmaceutics is limited due to its low erosion resistance and rapid degradation in human body fluids [16,17,18,19,20]. Such a quick and irrepressible corrosion mechanism leads to a notable reduction in the mechanical characteristics of the bioimplants, leading to early failure. Moreover, H_2_ gas production and the alkalinization of microenvironments near the interface of the tissue/ implant generates major concerns for the patient’s health [21,22,23,24,25]. Such negative influences might lead to rejection of treatment or even local tissue necrosis. Thus, monitoring the Mg corrosion/degradation rate is important to its clinical usage [26]. Scientists have concentrated on composite fabrication, surface engineering and mechanical alloying to increase corrosion resistance of Mg alloys [27,28]. Alloying is a notable approach for optimizing the characteristics and structures of materials but is also the primary factor for the high price of such materials [29]. Aside from alloying, surface-enhancing technology is an excellent tool to reduce the deterioration rate of Mg alloys [30]. Surface modification using different procedures—including electroless [31,32], electrochemical plating [28,33]; conversion coating [34,35]; physical vapor deposition [36,37]; plasma spray [38]; anodizing [39]; chemical vapor deposition [40,41]; and plasma electrolytic oxidation (PEO) [42,43,44,45,46]—are the most current methods for improving the characteristics of magnesium and its alloys. Amongst these methods, PEO is a developed approach that processes ceramic-like coatings. Coating using PEO with a pulsed source is an electrochemical surface therapy technique that generates a homogeneous oxide film in the plasma state by employing high voltage in an appropriate solution. The characteristics of the coatings (i.e., microstructure, composition, roughness and porosity) can be moderated via the electrical factors of the procedure and the composition of the solution [47,48,49]. The PEO of Mg has been demonstrated to be a good method for decreasing the rate of corrosion and preventing high alkalinization surrounding the tissues [50,51]. Several studies on PEO-formed coatings applied on various magnesium alloys have investigated the coating design (e.g., optimization in the composition of the PEO film and its sealing via sol-gel coating) and efficiency in vivo and in vitro, but various factors remain to be considered [52,53]. The effective surface area of the PEO-formed coatings increases as the number of pores increases, and the corrosive environment tends to attract and concentrate within the pores. This phenomenon will facilitate the penetration of the corrosive environment inside the coating segments and then towards the substrate, lowering the coating corrosion resistance and allowing H_2_ to escape [15,54,55,56]. To improve the protective properties of PEO layers on Mg alloys, several approaches have been developed. Forming an organic film on a PEO-layer surface is one of the most effective strategies for sealing microdefects and preventing aggressive species from entering the substrate. The effect of sealing components on improving the corrosion resistance of PEO layers by sealing cracks and pores has also been investigated. Some of the technologies used to seal the PEO-layer pores were phosphate, sol-gel, polymer coating and metals electroless deposition. Polymer coatings are of particular interest in the field of biomedicine because some polymers can be surface modified to allow the attachment of bioorganic molecules, such as lipids and proteins, significantly increasing the coating’s biocompatibility [57,58,59,60,61,62,63]. Polymer coatings are also easy to apply as a simple dipping-drying procedure can produce thick, protective coatings with minimal energy use [64]. Mg is not well-bonded with the polymer coating. Nevertheless, the PEO-formed coatings can serve as physical bonding sites or porous surfaces that are valuable in bonding the polymer coating. Therefore, different kinds of polymer coatings—such as poly (glycolic acid), collagen, poly (lactide–coglycolic) acid (PLGA), chitosan (CS), polycaprolactone (PCL), polylactic acid (PLA), alginate and fibrin—are used. In addition, polymeric materials have remarkable multifunctional properties, which could be utilized in subsequent Mg bioimplants (Figure 1). The latest developments in biocompatible and bioactive composite coatings are considered in this paper.

## 2. Properties of Mg and Its Alloys: Challenges and Solutions

Given their desirable mechanical properties and low density, Mg alloys are excellent choices for biodegradable implants [65,66,67,68,69]. The mechanical properties of Mg make it suitable as a biodegradable metal implant for orthopedic and stent uses. The difference in the elastic moduli of other engineering materials and the natural changes of the bone may lead to a stress-shielding effect between the injured bone and implant because such phenomenon adversely affects implant stability, preventing the bone under normal stress from harmonizing with the whole cure process [11]. In general, using degradable implants reduces the need for another operation to remove the implant. In addition, degradable implants stimulate the hurt tissue due to the slow transfer of load from the implant to the tissue [12,70,71]. Thus, an Mg implant presents implied advantages as a suitable material for a biodegradable implant.

Biodegradable magnesium was first used as a fixation tool to repair bone fractures. Nevertheless, several efforts had failed because of the fast corrosion of Mg and its production of subcutaneous bubbles and gas cavities, resulting in a great decline in mechanical strength. Mg has a high chemical function and can be degraded by a galvanic corrosion procedure [72,73]. The degradation of magnesium and its alloys basically requires the following reactions [74]:

Anodic reaction: Mg → Mg^2+^ + 2e^−^

Cathodic reaction: 2H_2_O + 2e^−^ → H_2_ + 2OH^−^

Overall reaction: Mg + 2H_2_O → Mg(OH)_2_ + H_2_ ↑

Mg(OH)_2_ accumulates on the underlying matrix of Mg, operating like a corrosion protective film within the water. However, this porous Mg(OH)_2_ layer will probably decline, especially in electrolytes with ions of chlorine as follows:

Mg(OH)_2_ + 2Cl^−^ → MgCl_2_ + 2OH^−^

**Figure 2:**
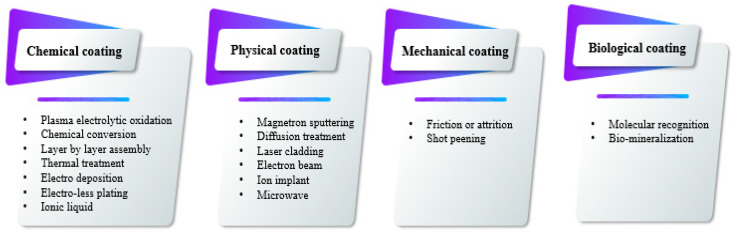
Schematic of coatings on the surface of magnesium and its alloys.

Therefore, the Mg(OH)_2_ film is not able to keep the Mg matrix safe from subsequent corrosion, and magnesium and its alloys are simply degraded due to the existence of ions of chlorine in the human body. Coatings can be deposited on the surface of magnesium through chemical, physical, mechanical, biomimetic or biological methods (Figure 2) [75]. According to target usage, the necessary rate of degradation and cell response, a coating can be formed on the surface of a magnesium biomaterial.

## 3. PEO Process

PEO is well-recognized as a unique type of surface modification technology that may be used to create ceramic coatings on metals, such as aluminum [76,77], magnesium [78,79,80,81], zirconium [82,83,84], Ti [85,86,87,88] and niobium [89], and their alloys. Electrochemical modification techniques for Mg alloys are divided into two classes, each of which is driven by a different voltage. The first class is a common anodizing process that operates in a voltage range smaller than the breakdown voltage. The formation of a natural passive layer in a certain electrolyte is accelerated in this technique, which is then fine-tuned by applying voltage. The second class works within a range that is higher than the breakdown voltage. Electrical discharge in the solution/metal interface is used in this process to improve the formation of film, optimize phases and the film chemical composition, as well as to generate a thick layer. High energy at the sites of electrical discharge can melt the passive film and substrate, transforming them into a hard ceramic coating with components of positioning solution. Electrical discharges are important aspects of the process because they are in charge of inserting components in solution into the expanding layer and sintering it to a thick ceramic-like film. Given that the sparks remain, a completely porous structure is achieved [61,90,91,92].

**Figure 3:**
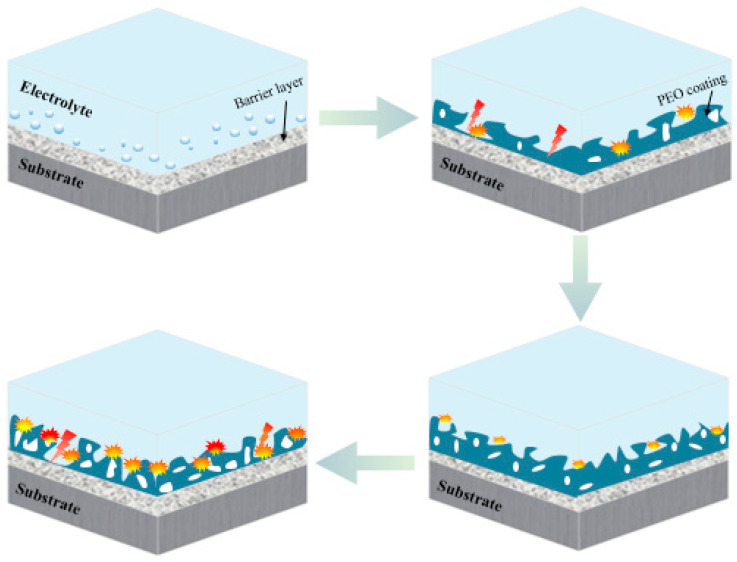
Schematic of PEO coating steps on a light-metal substrate [100].

**Figure 4:**
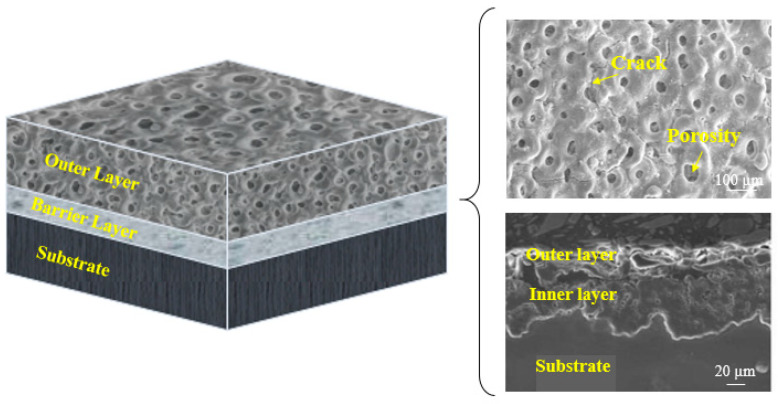
Schematic of distinct film for PEO coating [83].

Many variables, such as PEO settings and post-treatments, have been found to impress the PEO-formed coating quality. The effect of modifying the PEO parameters on the shape and other features of PEO-formed coatings on Mg alloys will alter the PEO discharge property. The qualities of such coatings are influenced by various factors, such as solution composition, substrate, electrical parameters, type of additive alloy, electrolyte temperature and duration of treatment [23,51,93,94,95,96,97,98]. According to Rakoch et al. [99], PEO has four main phases during the deposition of an oxide layer: spark discharges and/or anodizing, electrolysis, micro-arc discharges and arc discharges. As shown in Figure 3 [100], in the anodizing and/or electrolysis stage, in addition to the considerable rise in the anode voltage of the employed electrode, a fully porous coating is formed on the work-piece substrate. The introduction of a voltage that is comparable to the capacity of dielectric in the oxide layer causes a discharge in the following step (i.e., park discharges). With different geometrical flow intensities and sizes, the last two stages (i.e., arc and micro-arc discharges) occur simultaneously, resulting in the growth of deposition. Micro-arcs are applied on the electrolyte, rather than coating, in the final step due to the high release of energy in the arc discharges. Thus, local micro-sized flaws are found in the coatings, which are not desirable due to a deterioration in corrosion resistance [99,101]. The microstructure of the PEO-formed coatings is determined by the procedural conditions. Hence, coating thicknesses of 5–200 µm are possible. The undulating and uneven interface of coatings makes them a substrate intrinsic component. PEO-formed coatings, in particular, have a really thin barrier film, with thickness changing from several nanometers to 2 μm [102]. As the procedure time increases, the oxide film on the barrier film top continues to increase and thicken [103,104,105,106]. Some pores form and are situated in the coating as the ceramic layer is sustained by the electrical discharge process. The porosity amount of the PEO-formed coating is a function of the duration and the intensity of the electrical discharge. For several types of PEO treatments on magnesium alloys, pores with diameters ranging from 0.5 to 50 μm have been observed [107,108]. Images of SEM obtained from the PEO-formed coatings top surface display structures having uniform and fine pores or big and rough oxide scales having big pores on the surface (Figure 4).

## 4. Surface Properties of the Composite (PEO/Polymer) Coatings on Magnesium Alloys

Some coatings prepared with more than one layer based on the PEO are an effective surface modification approach to increasing the bioactivity and corrosion resistance of the biomaterial. An applied coating on the surface of the magnesium alloys does not imply great corrosion resistance, biodegradability, adhesion, bioactivity or other properties. Thus, multi-layer coatings on the biomaterial are developed. PEO has been used to prepare films with several types of polymer coatings. From the analysis of the adverse effect on the corrosion resistance of magnesium alloys, duplex coatings were found to perform better than single-layer coatings. Many polymer coatings have been used to protect magnesium biomaterials from corrosion.

The PEO-formed coating has a large quantity of microcracks and micropores that are created during electrical ignition in the coating. Such spatial flaws allow hostile ions to infiltrate the underlying substrate surface [109]. As a result, Mg substrates receive some protection, especially in the early stages. Other operations of pore sealing are needed. Hence, polymer coating might be a viable choice. The deposition of a biodegradable polymer layer is one of the solutions to address the long-term corrosion resistance of magnesium alloys in a physiological medium. To generate a better function, the coating should be devoid of porosity and should be uniform and biocompatible, possessing self-healing ability [26]. The type and thickness of the biodegradable polymer coating should be precisely chosen to adjust the degradation time of the coated substance.

The number and molecular weight of layers of polymer coatings have a significant effect on the structure of polymer-coated alloys of magnesium. The number of layers in a film can impact its density and thickness, and the molecular weight of a polymer can affect its rate of hydrolysis. Given the smooth surface and crack-free nature of CS, Gu et al. [110] showed that a six-layer CS coating formed 2.7 × 10^5^ molecular weight presence had the least rate of hydrogen development for Mg-1Ca alloys. CS will possibly take a longer time to degrade. CS is widely used in biomedical applications because of its bioactivity, biodegradability, biocompatibility and anti-microbial activity.

**Figure 5:**
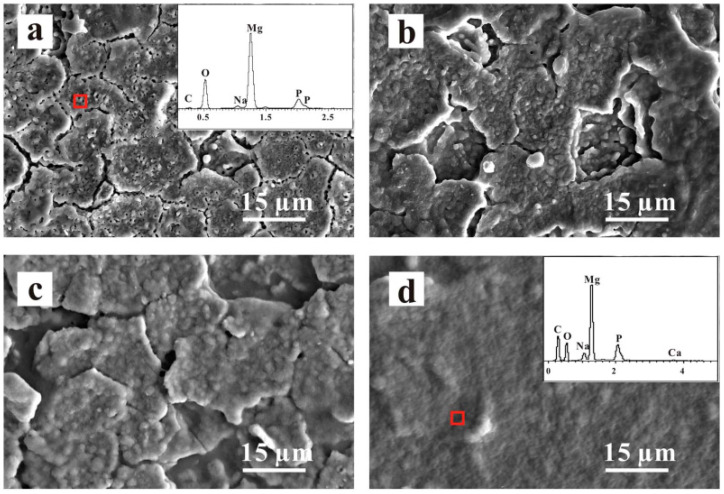
SEM images of the (**a**) PEO, (**b**) PEO/CS-1(0.1 g CS), (**c**) PEO/CS-2 (0.5 g CS) and (**d**) PEO/CS-3 (1 g CS) coatings [112]. (With permission from Ref. [112]; License Number: 5364041385585, 8 August 2022).

**Figure 6:**
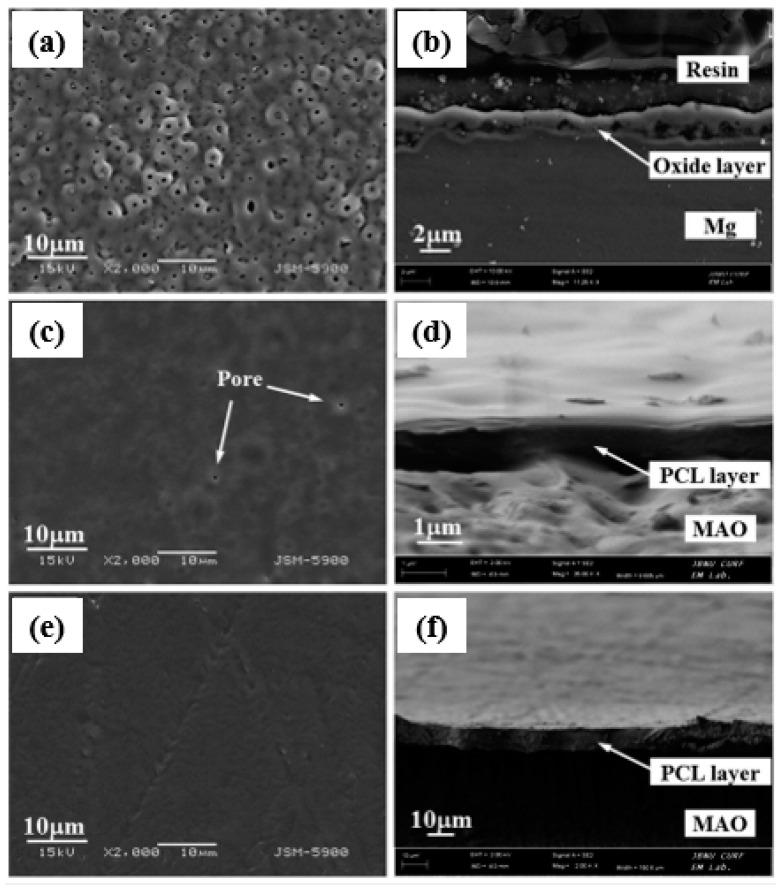
(**a**,**c**,**e**) Surface morphological and (**b**,**d**,**f**) cross-section images of the PEO-treated and PEO–PCL duplex-coated magnesium: (**a**,**b**) PEO-treated magnesium, (**c**,**d**) PEO–4PCL duplex-coated magnesium, and (**e**,**f**) PEO–7PCL duplex-coated magnesium [114]. (With permission from Ref. [114]; License Number: 5364050032518, 8 August 2022).

Similar findings have been observed by Dai et al. [111] on a composite of CS-coated Ca_3_(PO_4_)_2_/Mg-Zn. Amongst the 4-, 7-, and 10-layer CS coatings on the composite, the 7-layer CS coating had the best corrosion resistance. The 4-layer CS coating was extremely thin, whereas the 10-layer CS coating was extremely thick. The pores during PEO coating acted as polymer ‘interlocking’ sites. Yu et al. [112] investigated the biological behavior of a PEO composite coating on an Mg alloy with a CS polymer layer. The PEO-formed coating was used on an electrolyte containing phytic acid and NaOH. Thus, to apply a polymer coating, various concentrations (CS-1, 0.1 g; CS-2, 0.5 g; CS-3, 1 g CS) of the CS polymer were chosen and applied on the PEO-formed coating with a dip-coating technique. The generated PEO-formed coating was ~8 μm thick, whereas the CS polymer coating was ~3 μm thick. The findings showed that the PEO coating on the Mg–4Li–1Ca alloy had a typical morphological variation of cracks and pores, as can be seen in Figure 5a. The cracks and pores were larger than those found in the PEO coating on this alloy [113] since the previous contained more Li and had a lower ratio of Pilling-Bedworth than the latter. Micropores resembling volcano tops, ranging in size from 0.5 μm to 2 μm, were randomly spread on the surface (Figure 5b–d). In Figure 5d, the composite coating was sealed, and the layer surface became smooth. The EDS result, as illustrated in the inner portion of Figure 5a, showed that the PEO coating mostly consisted of magnesium, oxygen and phosphorus, indicating the existence of a phosphorus and MgO compound. P might have been derived from the phosphoric solution and the presence of more C. Li et al. [114] used an alkaline fluoride-based electrolyte for the PEO-formed coating and various concentrations (4 wt.% and 7 wt.%) of PCL to precipitate that the polymer coating on pure Mg determines the CS coating. PCL is regarded as a reliable and biodegradable polymer with a slow breakdown rate and has been used as a bioresorbable material. This multifunctional material will help seal the pores inside the PEO-formed coating and may even indicate the possibility of obtaining a desirable corrosion rate for magnesium and its alloys. Figure 6 exhibits the cross-sectional pictures and floor morphology of the PEO-formed coating with Mg and the Mg coated with PEO–4PCL and PEO–7PCL duplex. The Mg treated with PEO had a regular crater-like shape with micropores having a diameter of 1 μm (Figure 6a). The cross-sectional view in Figure 6b indicated the formation of a stable coating with a thickness of 2–3 μm and a thin barrier film. The PEO–4PCL duplex-coated surface revealed the formation of a constant polymer layer that was consistently covered over the crater-like PEO-treated surface with only a few micropores (0.5 μm; Figure 6c). Given the lower PCL content (4 wt.%) and the number of coating cycles employed, flaws, such as micropores, could not be completely avoided. The thickness of the PCL coating was as thin as 600 nm, revealing the shape of the PEO-treated Mg substrate (Figure 6d). Coatings made with electrolytes containing less than 4 wt.% PCL lack consistency. For the PEO–7PCL duplex-coated Mg, the crater-like morphology was not visible on the surface, allowing for the creation of a stable coating with no pores having a flawless coating over the PEO-treated Mg (Figure 6e). The cross-sectional view confirmed the stability of the coating and revealed that the coating was rather thick (on the order of 9 μm; Figure 6f).

Given the high viscosity of the electrolyte, the coating lost consistency when the PCL concentration was increased to more than 7 wt.%. The concentration of the PCL coating electrolyte and the number of cycles of the dip-coating may be used to monitor the quality and thickness of the PEO–PCL duplex coating to determine the rate of biodegradation of the Mg implants inside a biological medium.

Yu-Kyoung et al. [115] studied composite coatings for biodegradable applications by using Mg screws with PCL polymer. First, pure Mg was coated by PEO on electrolytes containing Na_3_PO_4_ and NaOH under pulsed current. In the second stage, electrolytes with different concentrations of PCL (5 wt.%, 6 wt.% and 7 wt.%) were provided. Mg screws coated via PEO were immersed within a polymer electrolyte by using the dipping technique. The immersion of the specimens was repeated using various (2, 4 and 6) cycles. The surface of the PEO-coated Mg screws augments the surface hydrophilicity and area, producing a steady and stable PCL polymer film on the surface with good adhesion. Thus, a polymer layer was produced on the PEO film by changing the number of coating cycles and PCL concentration. The results indicated that 7 wt.% of PCL and twice the number of cycles generated thick coatings. Although the high thickness enhanced the protective properties, such a process will result in the coherent failure of the coating if it is more than the optimal amount. Moreover, the steadiness of the coating is significant. In this case, the upper part of the screw was entirely coated, but the thread part was not coated by a polymer. Thus, by increasing the PCL concentration and the number of cycles, the stability of the coating was amended, and the Mg screw is wholly coated. Such characteristics enhanced the biocompatibility properties of the coating.

Santos-Coquillat et al. [116] used the figures of breath method (BF) for producing a porous film consisting of PCL on a PEO-treated Mg alloy surface for increasing tissue vascularization. The BF method involves the combining of a polymer having a highly hazardous organic solvent and evaporating the polymeric electrolyte within a wet medium [117]. The latest research has indicated that the usage of polymers closes the pores and defects of the PEO-formed coating, thereby increasing its degradation in body simulated environments. Based on the results and to obtain the best properties, the polymer weight percentage, the number of films and the thickness of the coating should be regarded in the optimal situation.

## 5. Biocorrosion Behavior of the Composite (PEO/Polymer) Coatings on Magnesium Alloys

A polymeric coating was employed with the PEO coating of magnesium alloy to produce a hierarchic porous structure that may change the bioactivity and provide further corrosion protection. A top polymeric film decelerates the degradation of magnesium and its alloys [118] (Corrosive species should diffuse towards the Mg substrate through an extra film, resulting in gentler corrosion.) and ensures the longer structural integrity of the tool. Such characteristics ascertain that the reconstruction plate does not break prematurely. However, just applying a polymeric coating to an uncoated alloy is insufficient to prevent primary alkalization and maintain a monitored rate of deterioration [30,119].

Biodegradable polymer coatings on magnesium alloys can boost the long-term corrosion resistance of implants in the body and the biocompatibility of metallic Mg in biodegradable applications [120,121,122]. Natural biodegradable polymers, including collagen and CS, and synthetic biodegradable polymers, including PCL and poly–L–lactide (PLLA), provide enough fortification and have been shown to be biocompatible with Mg and its alloys [123,124,125]. CS has been demonstrated to be biodegradable, biorenewable, biocompatible, non-antigenic, non-toxic and biofunctional. This material is composed of glucosamine and N-acetyl glucosamine units connected by one to four glycosidic linkages [126,127]. Natural polymers are more biocompatible, mostly because of the absence of highly acidic degradation items compared with synthetic polymers [128]. CS has been employed to improve magnesium and its alloys to enhance their biocompatibilities and corrosion resistance. Furthermore, the barrier function of the CS coating may permit the biodegradation of the Mg implant at a desirable pace. For the Mg Zn–Ca alloys, a comparison of a single PEO film versus a CS film revealed the superiority of the PEO/CS composite coating corrosion resistance [129]. This impact has been attributed to the barrier properties and pore sealing of CS. Nevertheless, the improvement in corrosion performance is still restricted. In addition, the mechanism of deterioration of the PEO/CS layer applied to magnesium alloys has not been determined. Thus, self-recovery or self-healing is a significant category of smart coatings that are significantly considered in material science [130]. A crucial feature of the CS for extensive use in Mg alloys is its capability to mend the coating structure. The self-healing ability of a polymer can be obtained via a couple of different strategies, namely, the microcapsule strategy and stimulus-responsive healing procedure [131].

**Figure 7:**
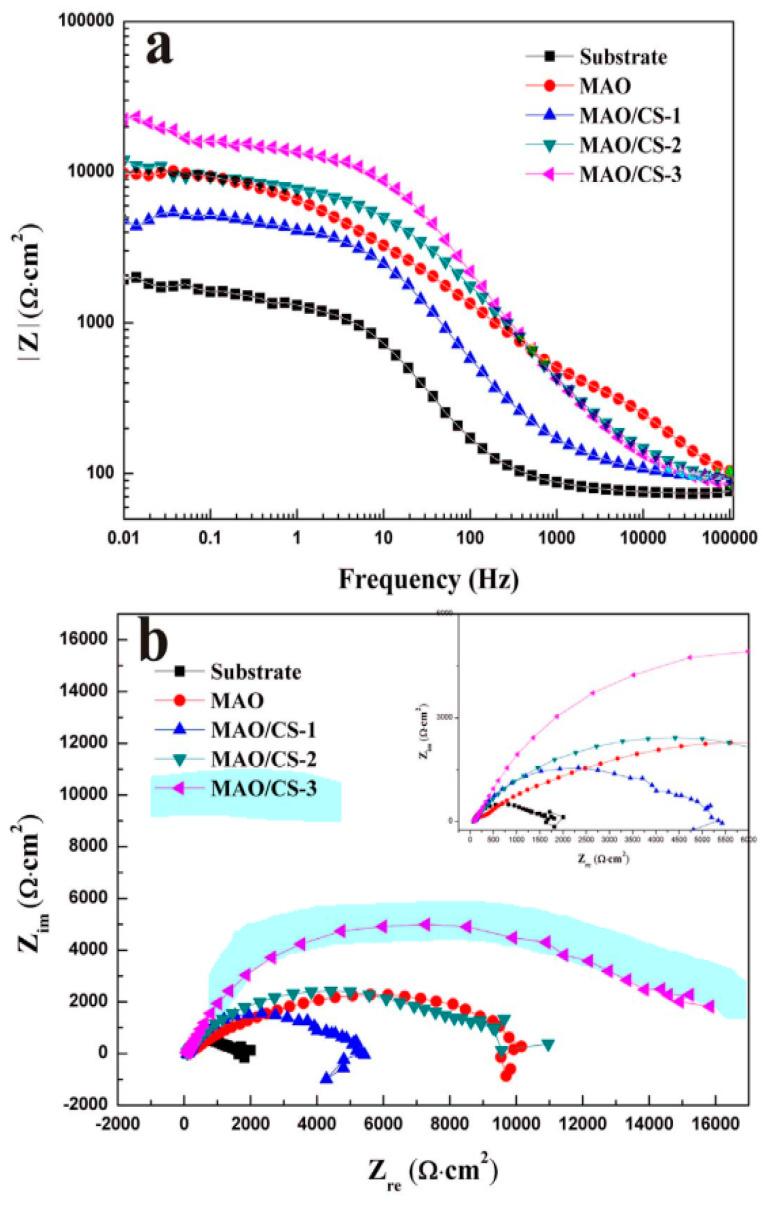
(**a**) Bode and (**b**) Nyquist curves of the Mg–4Li–1Ca alloy and coatings in Hank solution (CS-1, 0.1 g; CS-2, 0.5 g; CS-3, 1 g CS) [112]. (With permission from Ref. [112]; License Number: 5364041385585, 8 August 2022).

**Figure 8:**
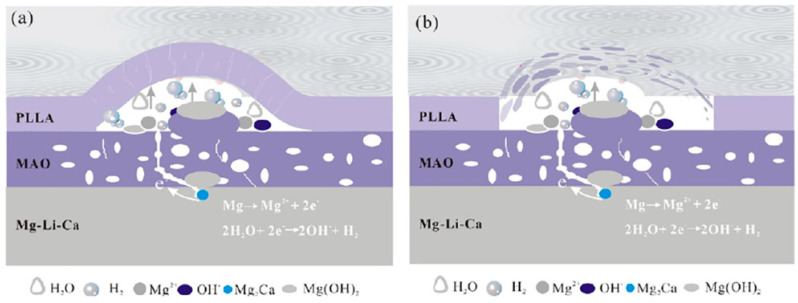
Schematic of degradation mechanism of a PEO/PLLA composite coating on Mg alloy in Hank solution: (**a**) swelling of PLLA and corrosion of Mg alloy at the initial stage, (**b**) blistering and final peeling-off of PLLA under corrosion products and hydrogen gas pressure [113]. (With permission from Ref. [113]; License Number: 5364050148432, 8 August 2022).

**Figure 9:**
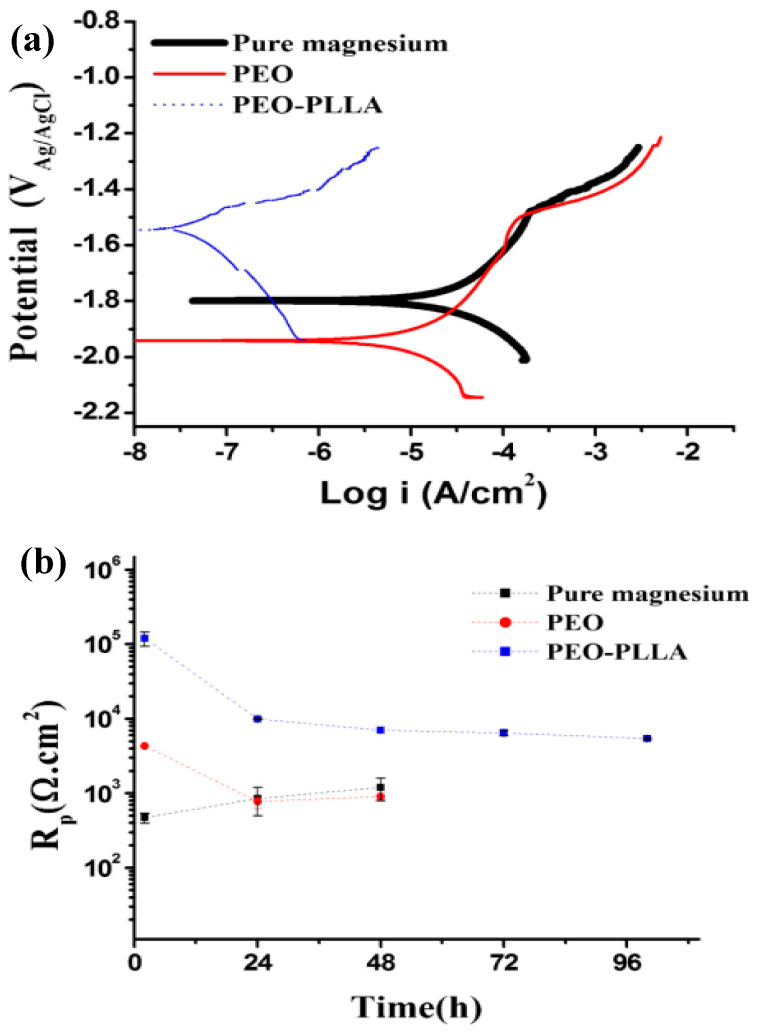
(**a**) Polarization plots of Mg, PEO-coated and PEO–PLLA-coated specimens in SBF after 2 h immersion, (**b**) Polarization resistance (R_p_) of pure Mg, PEO-coated and PEO–PLLA-coated specimens in SBF after different immersion intervals [142]. (With permission from Ref. [142]; License Number: 5364050271809, 8 August 2022).

Jia et al. [132] employed a PEO-formed layer to introduce Ce to CS into an Mg–Ca alloy. Nanoparticles were used to create a unique stimulus-responsive self-recovery mechanism. The inhibitor Ce III was chosen as the carrier, and the polysaccharide CS was used to provide the micro-nano “reservoirs” unique structure for CS + Ce. A biocompatible multi-layered ceramic/poly-electrolyte anti-corrosion system was established by the spin-coating and dip-coating processes. The prolonged delivery and severe particle immobilization were attributed to the coordination of Ce–NH_2_ between the CS and the inhibitor. Thus, the protective properties of the covering were demonstrated. The PEO–CS coating relatively reduced the substrate high-frequency impendence modulus, most likely due to the CS bulge that filled those micro-spores/defects, and adding the cerium inhibitors resulted in active self-healing behavior. As previously indicated, the concentration of the polymer coating, coating density and thickness have a major effect on the corrosion behavior of Mg alloys. Yu et al. [112] studied the corrosion of CS polymer films at three different concentrations. EIS was performed to assess the structural characteristics and corrosion resistance of the coatings (Figure 7). For the metal substrates, a larger radius of curvature and a greater Z modulus at lower frequency are commonly assumed to indicate better corrosion resistance [133]. The specimens were of the following order as shown in the Bode plots (Figure 7a): PEO/CS-3 > PEO > PEO/CS-2 > PEO/CS-1 > substrate PEO/CS-3 > PEO/CS-2 > PEO/CS-1 > substrate. The Nyquist diagram (Figure 7a) also indicated that the PEO/CS-3 specimen had the largest radius of curvature, indicating that it had the best corrosion resistance. As a hydrophobic aliphatic polyester having recyclable resources, PLA is suitable for biomedical uses [134]. This material emphasizes the importance of biocompatibility, biodegradability and thermoplastic processability [135,136,137] and has been applied in some useful medical gadgets [138,139]. Lu et al. [140] recently explored the in vitro degradation of PLLA dip-coated on PEO-treated Mg alloy of WE42. Compared with the PEO-treated Mg alloy, a 40% rise in the primary polarization resistance (Rp) was observed for the coated alloy of PEO–PLLA. The dip-coating process, in general, produces a reasonably thick coating with excellent primary resistance. Nonetheless, given that PLLA tolerates bulk deterioration, long-term exposure of a thick covering might result in its peeling [141]. Zeng et al. [113] employed dip-coating followed by freeze-drying to create a PEO/PLLA composite coating on the alloy of Mg–1Li–1Ca for orthopedic usage (Figure 8). Outcomes showed that the composite coating of PEO/PLLA considerably boosted the corrosion behavior of Mg–1Li–Ca alloy. Nevertheless, liberation of H_2_ augmented the interior stress under the PEO and coating of PLLA, reducing the coating mechanical properties. Alabbasi et al. [142] investigated the corrosion performance of a PEO film sealed by the PLLA polymer on pure Mg. The curves of polarization related to the PEO-formed coating showed pure Mg and PEO–PLLA-coated specimens as indicated in Figure 9a. Compared with that of pure Mg, the corrosion current density (i_corr_) of pure magnesium was declined by 65% with the PEO-formed coating and by almost 100% with the PEO–PLLA coating, according to the cathodic curves. Also, the E_corr_ of the PEO–PLLA-coated specimens shifted by 250 mV to the noble direction, whilst the samples coated using PEO shifted by 100 mV to the active direction. The results show that the PLLA coating filled the pores of the film produced by PEO, preventing the solution from penetrating. As a result, the Rp of the PEO–PLLA-coated specimens was greater than that of the PEO-coated specimens throughout the measurements. The uncoated and coated metals were subjected to long-term in vitro degradation tests. Figure 9a shows the Rp values for the specimens at various exposure durations inside the SBF. After 2 days, the Rp of the PEO-coated specimens fell by 80%. After a 24-h exposure, the Rp of the coated PEO–PLLA specimens declined. Nonetheless, this Rp value was more than one order of magnitude larger than those of pure Mg and PEO-coated specimens. The Rp for the PEO–PLLA specimens was determined to be 5.4 × 10^3^ Ω·cm^2^ even after 100 h of exposure, which was remarkably greater than the Rp for the PEO-coated specimens after 2 days. The outcomes showed that the PLLA coating filled the holes in the PEO film and prohibited the electrolyte from penetrating. Lu et al. [140] reported an Rp for a PEO–PLA-coated alloy that was higher in comparison to what we investigated in our research because of the difference in the pH state of the solution between measurements. If the pH of the solution is not adjusted normally within long-term experiments, such as is the case in Lu et al. [140], magnesium passivates and the Rp are not significantly modified or even rises. Thus, changing the SBF on a regular basis throughout long-term testing is critical to keep the pH of the solution close to the physiological level. To produce a PLA/PEO layer on AZ31, Shi et al. [143] employed PEO combined with PLA sealing. PEO was performed in a solution having 4 g/L KF and 40 g/L NaOH. A porous MgO film having a thickness of ~5 μm was deposited on AZ31 by using PEO, and PLA sealing was performed using dip coating having a thickness of ~9 μm. The acquired bonding strength between the layer and the underlayer was 45 MPa, which was higher when compared to the obtained adhesive strength between the underlayer and the PLA coating. To show the mechanics of bonding at the coated layer interfaces and substrate, this investigation offers a mechanical interlocking and MgO chemical bond model. In the SBF, the PLA coating can dramatically improve the anti-corrosion properties of the uncoated alloy and AZ31/PEO. The PEO–AZ31 and PLA–AZ31 corrosion potentials were favorably altered by ~110 mV in comparison to that of the AZ31 substrate. From the uncoated AZ31 (611.71 A/cm^2^) to PLA–AZ31 (7.72 A/cm^2^) and PEO–AZ31 (66.32 A/cm^2^), the corrosion current density decreased. Although the PLA–PEO–AZ31 corrosion potential was higher (1.511 V), the corrosion current density was substantially lower. This result indicated that the impressive sealing of the PLA film prevented the corrosive ions from entering the vents and fractures, thereby raising the corrosion resistance of the MgO coating. Surface modification of the magnesium alloys—to delay their rate of deterioration in the SBF with PEO/PLA composite coatings with strong corrosion resistance and adhesive strength—is an appealing solution. Bakhsheshi Rad [144] found that a PLA film may be formed on a PEO-coated Mg–Ca alloy by using a very easy process. Given the composite coating, the contact angle increased significantly from 42.30° for the naked alloy to 95.30° for the duplex coating. The bonding strength results showed that the PEO coating had a good bonding strength (23.5 MPa), whereas the PEO/PLA coating had a lower bonding strength (14.7 MPa). However, when compared to the bonding strength of the direct PLA coating on the surface of Mg alloy without PEO treatment, which was only 2.5 MPa, the PEO/PLA coating demonstrated significantly higher bonding strength. When compared to the single-film PEO coating, the double-film PEO/PLA coating had a remarkably greater corrosion current density and a better charge transfer resistance. Their findings also revealed that enclosing the pores in the PEO film with PLA polymer coating significantly improved the magnesium alloy corrosion resistance in the physiological medium of SBF, indicating the potential of the PEO approach for biomedical applications. Despite these advantages, PLA has drawbacks when it comes to the proliferation of biodegradable magnesium alloys in orthopedics. The biggest disadvantage of PLA as a barrier film is its eagerness to hydrolyze in water [8].

With the breakdown of magnesium alloys, the PLA coating bulges because of the release of H_2_ gas. This phenomenon results in the peeling off of holes and fissures in the PLA coating, finally resulting in distortions [145]. Thus, further improvement in the preparation and design of PLA as a barrier coating for magnesium-based alloys is required.

PCL is a biodegradable polyester [146] with excellent Physico-chemical qualities, including biodegradability [147], biocompatibility [148], structural stability [149], low melting point and flexible mechanical function [150]. PCL has two types of degradation, namely, hydrolytic and enzymatic [151], which produce oligomers with carboxyl groups. Hydrolytic degradation happens largely in the material bulk, while enzymatic degradation promotes erosion [152]. Moreover, the friendly characteristic [153], simplicity of production and tuneable qualities of PCL make it a compelling candidate for bone graft replacement [154]. Another advantage of PCL is its ability to prevent gas from forming on the base metal and to boost the interface between the implant and the bone. Thus, PCL is considered an appropriate coating material for controlling the pace of deterioration and mechanical strength. Despite these advantages, several researchers have added a single PCL coating to magnesium alloys to boost their resistance against deterioration. PCL is a biopolymer made from the ring-opening polymerization of the monomer ε-caprolactone9. This polymer degrades more slowly than PLLA and has been used as a surface coating on Mg alloys. Kim et al. [115] studied the corrosion resistance of a polymer covering on a PCL Mg screw. In addition, PEO was used to improve the adherence of the polymer to the metal. However, when a complex item, such as a screw, is inserted in the bone, the surface coatings are locally harmed, and the protective role of the coating is not well maintained. The ideal conditions for the creation of a polymer coating on screws were determined in this study by varying the PCL concentration (5 wt.%, 6 wt.% and 7 wt.%) and coating (2, 4 and 6) cycles. In general, the surface tension and viscosity of an electrolyte are proportional to the amount of PCL present in the solvent. If the viscosity is also low, generating a layer with a thickness more than a predetermined restriction will be difficult. If the viscosity is too high, a coating with non-uniform thickness will be formed on the curved surface of the screw. By increasing the entanglement degree, the coated electrolyte viscosity increases. On all surfaces, the coating formed with 6 wt.% PCL and 4 cycles was consistently applied. After three months of immersion in the SBF solution, the findings showed that fractures in the polymer film had formed, and the solution had permeated into the porous PEO-coated film. These events caused corrosion in the Mg metal and a rapid increase in pH. Furthermore, Mg corrosion products that accumulated under the PCL coating generated and collected H_2_ gas, causing the PCL coating layer to inflate and eventually disappear.

Glycolic acid (GA) and Lactic acid (LA) copolymerize to form PLGA [121,155]. Changes in the ratio of the two co-monomers can be used to manipulate the mechanical, physical and chemical characteristics of PLGA [8]. PLGA possesses a great function as a coating on alloys of magnesium, with the potential to adjust the rate of deterioration. One of some synthetic polymers that have been confirmed to be utilized in human clinical trials is the PLGA co-polymer, which has outstanding biocompatibility. Such material is also biodegradable by hydrolyzing the LA and GA ester linkages that are normally excreted by normal metabolism.

**Figure 10:**
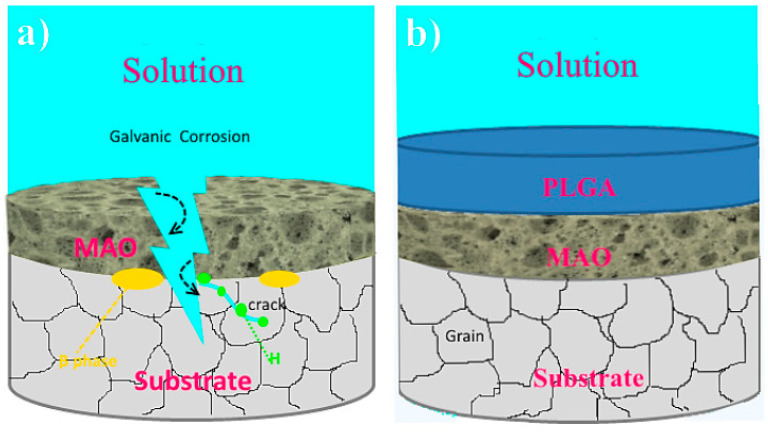
Schematic of the corrosion behavior for (**a**) PEO; (**b**) PEO + PLGA coated [156].

Chen et al. [156] used a PEO + PLGA composite coating on the alloy of Mg–4Zn–0.6Zr–0.4Sr. As shown in Figure 10, the PEO + PLGA coating considerably boosted corrosion resistance, stress corrosion cracking (SCC) and mechanical stability over time. Comparing to uncoated alloy of Mg, the PEO + PLGA coating increased corrosion performance by roughly 3 orders of magnitude, and SCC susceptibility tests declined by 75% of ultimate tensile stress and 50% of strain to failure. After 2% of plastic deformation, the PEO-formed coating failed, as shown by a change in values of the open circuit potential in the slow strain rate tensile tests. However, the PEO + PLGA composite coating prepared effective protection against more than 10% plastic deformation. For strained Mg alloy implant materials, the ductile PLGA had been anticipated to be a deterrent coating. Many studies have highlighted the use of polypyrrole (PPy) on Mg alloys for biomedical purposes amongst the investigated polymeric coatings because of its many qualities, such as high electrical conductivity, high stability, redox properties, facile manufacturing and adequate biocompatibility. Hatamin et al. [157] used an electrochemical method to deposit PPy layers on an AZ31 Mg substrate. The results showed that the passivation pretreatment improved corrosion performance and the surface of magnesium alloy adhesion strength. Arrabal et al. [59] applied polymeric coatings on the plasma-anodized AZ31 and linked the finding to a fluorocitrate–zirconate pretreatment layer. In comparison to the polymer coating of Ti/Zr, their findings depicted that the PEO + polymer coating provided great adhesion, appropriate impact resistance and increased corrosion resistance [59]. The effect of polymer coating on the corrosion behavior of PEO coatings is conveyed below in addition to Table 1 [112,114,115,142,158,159,160].

## 6. Biocompatible Behavior of the Composite (PEO/Polymer) Coatings on Magnesium Alloys

Although polymer coatings alone may not be sufficient to address practical objectives, mixing ceramics with polymer coatings is a promising path for biological uses. Implant-related infections, in addition to the relevance of corrosion function in the human environment, are major causes of implant failure in clinical settings with substantial medical expenditures. Therefore, using polymers with antibacterial characteristics increases the biocompatibility of implants. CS is a naturally occurring polymer with antibacterial attributes. This material is a common and natural substance that is made via treating the chitin shells of shrimp and other shellfish with an alkaline material, such as NaOH.

**Figure 11:**
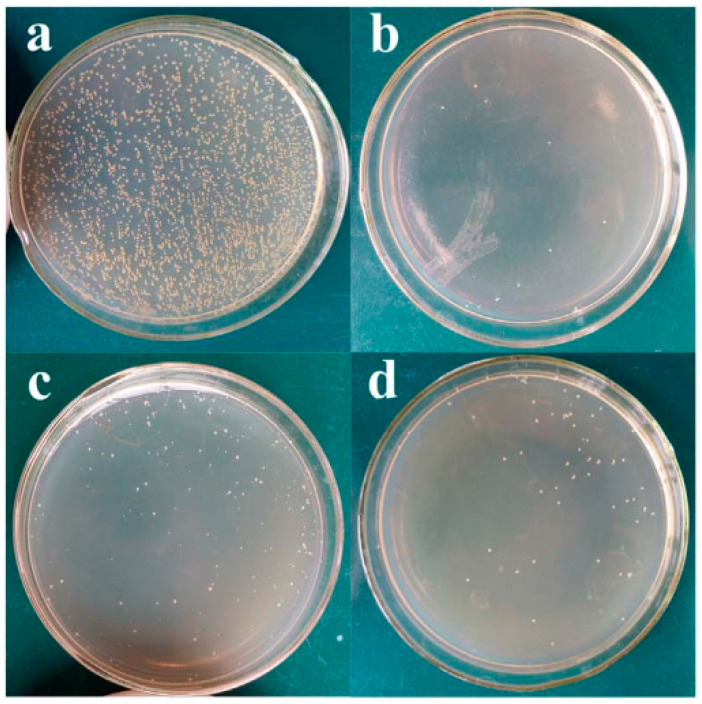
Photos of the growth of colonies on agar plates cultured with S. aureus for (**a**) blank, (**b**) Mg–4Li–1Ca, (**c**) PEO and (**d**) PEO/CS-3 [112]. (With permission from Ref. [112]; License Number: 5364041385585, 8 August 2022).

Numerous investigations, including those on the corrosion behavior and antibacterial performance of CS on magnesium and its alloys, have been conducted. Yu et al. [112] studied the antibacterial activity of polymer films with different CS concentrations against *S. aureus*. The plate-counting approach was used to assess the antibacterial properties of the uncoated and coated Mg alloys versus *S. aureus* (Figure 11) [161]. The PEO coating increased the count of CFUs to 253 when a diluted specimen from the control tube (the alloy of Mg 4Li–1Ca) was plated. This phenomenon could be attributed to the high pH (9.3) of the magnesium alloy, preventing bacterial growth [161]. Thus, the CFU count in the PEO/CS-3 specimen dropped to 89, indicating that the anti-bacterial action was considerably enhanced by the CS coating. Given the high pH produced by the dissolution of the bare Mg alloy, the uncoated Mg alloy demonstrated higher antibacterial activity in contrast to the PEO/CS coating (~ pH 9.3). Robinson et al. [162] discovered that increasing the pH from pH 7.4 to pH 10 improved the antibacterial action. Although the high pH provided greater antibacterial protection, the medium was not an appropriate implant medium. The PEO/CS coating had a superior antibacterial action because of the CS coating applied according to the contact-killing approach and a pH of ~8.4 that provided a proper implant setting for cell development and proliferation. Furthermore, the high levels of CFU used in this investigation are seldom found in real-world systems. Therefore, the composite covering is effective in inhibiting bacterial growth. Vancomycin (Van)-loaded sodium alginate (SA) hydrogel coating over PEO-treated Mg alloy may be further crosslinked by calcium ions (Ca^2+^), according to Minting et al. [160]. The antibacterial activity of modified Mg substrates against *E. coli* and *S. aureus* was investigated in this study. The original Mg and Mg/PEO substrates have almost no inhibition zone area to either *E. coli* or *S. aureus*, indicating that they have little antibacterial activity against either *E. coli* or *S. aureus*. Mg/PEO/SA–Van and Mg/PEO/cross-linked SA–Van substrates, on the other hand, showed a clear inhibition zone against *S. aureus*. The results showed that the Van released from the SA hydrogel coatings had a better bactericidal effect on *S. aureus* than on *E. coli*.

Cell adhesion, in addition to corrosion and antibacterial activity, is the most critical phase in the contact of a cell with a biological substance because of its significant impact on other cellular processes. Cells on bare samples are unable to adhere to the surface and spread. Several studies have found that the porosity on the surface of PEO coatings causes cell adhesion [163,164,165]. The pores on the PEO coating are sealed with a biocompatible polymer coating. Santos-Coquillat et al. [116] created a smooth composite coating by covering PEO-formed porous coatings with a PCL polymer coating. To stimulate tissue vasculature, they employed the BF figure to build a porous film formed using PCL over a PEO-treated surface of Mg alloy. The development of a porous polymer layer in this matrix might be an additional essential characteristic. The micrometer-sized holes in the porous material released the nutrients and oxygen required for cell survival and proliferation, prompting the cells to multiply. This approach was according to the evaporation of a polymeric electrolyte in a wet medium by means of a volatile solvent. As the solvent evaporated, the temperature at the solvent–air contact dropped, and water vapor condensation occurred. This process resulted in the production of ordered water droplet arrays at the solution–air interface [117,166]. The topography of the surface of BFs has been demonstrated to impact cell proliferation, migration and differentiation. The findings showed the interactions of cells with the coating system at several levels, eventually achieving the PEO structure at the bottom of the pores, where bioactive components may be accessed. Cytotoxicity refers to a substance’s toxicity to cells, and a cytotoxic compound can cause cell damage or death [167]. In this regard, they investigated the cytotoxicity of PEO/PCL coatings on Mg alloy substrates. A pre-myoblastic cell line was seeded over the materials, and cells were able to adhere (after 24 h) and proliferate in all of the evaluated materials (96 h). As a result, pre-myoblastic cultures adhered and proliferated without cytotoxicity over planar Mg–PEO–PCL and microstructured counterparts. As a result, PEO/PCL-coated Mg degradation can be tailored to promote cell adhesion and cytotoxicity. PEO/Polymer coatings that do not induce cytotoxic responses in cells are proposed as a long-term prevention tool to avoid bacterial attachment and proliferation.

Wei et al. [168] investigated the hemocompatibility, surface biocompatibility and corrosion resistance of a hybrid coating that was applied to an AZ31. For cardiovascular stents, PEO/PLLA, PEO/PLLA/PDA and PEO/PLLA/PDAM/Hep (heparinization) were investigated. The PEO/PLLA composite coating demonstrated the highest corrosion performance in Mg alloys, but the PDA adjustment and additional heparinization method provided contrasting results. All coatings had no discernible cytotoxicity, showing that they were safe for modifying Mg-based stents. Adding PDA to the PEO/PLLA composite coating is a promising method for biodegradable Mg-based implants for cardiovascular stents and orthopedic applications. Lu et al. [169] coated Mg alloy AZ81 using PEO and then immersed the alloy in PLLA electrolyte. The PEO/PLLA coating boosted the corrosion resistance of the underlying magnesium segments. Furthermore, a paclitaxel (PTX) electrolyte and a PLGA electrolyte were mixed and subsequently dropped on the PEO/PLLA-coated surface. The blood compatibility of the changed specimens was excellent.

Following the success of in vitro tests, in vivo tests are critical in the development and implementation of implants for use in the human body. The goal of these tests is to place the biomaterial near a living physiological system to examine its biocompatibility with tissue in an animal body to see if it is harmful to the body. Kim et al. [115] investigated the in vivo behavior of a polymer coating on a PCL Mg screw. Different conditions would be required to form a uniform PCL coating on a commercial device with a complex shape. The PCL coating produced under optimal conditions on a Mg plate was applied to the PEO Mg screw, with the goal of determining the optimal number of cycles (2, 4 and 6) and concentration (5, 6 and 7 wt.%) for producing uniform PCL layers on complex materials by conducting in vivo studies in a rat tibia. Stable and compact new bone formed on the PCL coating, as seen in the histological analysis results.

## 7. Conclusions

Mg-based biomaterials offer great potential for orthopedic applications because of their biodegradability and biocompatibility. Furthermore, the need for innovative biodegradable biomaterials comprising Mg and its alloys with new biological uses has been increasing. Biocompatible Mg alloys have been proven to be biodegradable and acceptable for usage in the body. Nevertheless, their use is restricted due to their low corrosion resistance. High-concentration corrosion products might also compromise the biocompatibility of this biomaterial. As a result, regulating Mg-based biomaterials in vivo is a difficult task. The obstacles that Mg biomaterials encounter were effectively overcome by biological surface modification of Mg alloys. A single coating process cannot generate a wide range of qualities. Thus, novel composite coating techniques for the advancement of biodegradable Mg-based orthopedic bioimplants with suitable bond fortification, high corrosion resistance and acceptable biocompatibility should be developed. Ceramic coatings generated by PEO have been proven to be a potential method to boost the characteristics of Mg alloys. The formation of a porous surface as a result of the ignition process is one of the key characteristics of PEO-formed coatings.

Although such porosities may not provide long-term protection for magnesium and related alloys, they can help the top coat to adhere better. Therefore, various polymer coatings have been developed. While polymeric coatings alone may not be sufficient to meet practical needs, PEO/Polymer composite coating is a promising alternative for certain application fields. This coating can solve problems by properly sealing defects. Long-term tests show that composite coatings have a longer service life, higher corrosion resistance, and bioactivity and biocompatibility. The main reasons for this two-layer system’s appropriate protection are the PEO coating’s adhesion to the substrate and the polymer coating’s high density. Filling the pores in the PEO structure allows corrosive ions to pass through to the surface. Improved corrosion protection using polymer coatings has been depicted in a lot of investigations. These coatings have been widely used due to their high chemical resistance, protective properties and high adhesion strength. Studies have shown that Mg coatings and alloys, including natural and synthetic polymers, can provide valuable features, such as corrosion resistance, bioactivity and cell compatibility. This review paper discussed how polymer coatings can be used to limit the deterioration of Mg alloys. Composite coatings on Mg-based materials offer a wide range of activities, including biocompatibility, bone induction, cell compatibility and antimicrobial properties. In a wide range of biomedical applications, PEO/polymer composite coatings will continue to be improved and created. Overall, the PEO/polymer coating system can be used in the estimated usage of PEO/polymer duplex coatings in the medical, electronic, aerospace, sports and leisure and auto industries.

## Figures and Tables

**Table 1:** Influence of polymer layer on corrosion properties.

Substrate	Polymer Layer	Corrosive Media	Time	*E*_corr_ (V)	*i*_corr_ (A.cm^−2^)	*R*_p_ (Ω.cm^2^)	Ref.
Mg-4Li-1Ca	Chitosan	Hank’s solution	13 min	−1.93	6.71 × 10^-6^	-	[112]
Pure Mg	Polycaprolactone (PCL)	SBF	3 months	-	-	-	[115]
Pure Mg	Poly(l-lactide) (PLLA)	SBF	2 h	−1.54	3 × 10^−8^	10^5^	[142]
Pure Mg	Polycaprolactone (PCL)	HBSS	7 days	−1.53	81 × 10^–8^	9.9 × 10^3^	[114]
Mg–Mn–Ce	Polytetrafluoroethylene (SPTFE)	3 wt.% NaCl	30 min	−1.50	5.4 × 10^−9^	1 × 10^7^	[158]
WE42	Gelatin	Hank’s solution	-	−1.42	9 × 10^–7^	-	[159]
AZ31	Polyethyleneimine (PEI)	SBF	-	−0.31	3.34 × 10^−7^	-	[160]

## Data Availability

Not applicable.
